# Prevalence and Correlates of Stability and Change in Maternal Depression: Evidence from the Fragile Families and Child Wellbeing Study

**DOI:** 10.1371/journal.pone.0045709

**Published:** 2012-09-20

**Authors:** Kristin Turney

**Affiliations:** Department of Sociology, University of California Irvine, Irvine, California, United States of America; Wayne State University, United States of America

## Abstract

Children of depressed mothers have impaired cognitive, behavioral, and health outcomes from infancy through adulthood, and are especially at risk when maternal depression persists over multiple years. But there are several important limitations to our current descriptive knowledge about maternal depression, especially depression among unmarried mothers. Data from the Fragile Families and Child Wellbeing Study, a recent cohort of children born in urban areas to mostly unmarried parents (N = 4,366), was used to examine the prevalence and correlates of maternal depression when children were about 1, 3, 5, and 9 years old. Results show that, at any given survey wave, between 16% and 21% of mothers reported depression. Nearly two-fifths (38%) of mothers reported depression at least once during the eight-year period, and 7% reported persistent depression (depression at three or four of the four survey waves). Employment status, relationship status, and fathers’ depression were among the sociodemographic characteristics most robustly associated with both stability and change in maternal depression. Given the important social consequences of maternal depression, not least of which is impaired wellbeing among children of depressed mothers, prevention and treatment of maternal depression should be an imperative for researchers, clinicians, and policymakers alike.

## Introduction

Mental health problems are an important public health concern. Major depressive disorder is one of the most common, chronic, and debilitating mental health disorders, affecting more than 13 million individuals annually in the United States [1]. The social consequences of depression are far-reaching, and the World Health Organization (WHO) lists depression as one of the most burdensome diseases in the world [2]. Virtually all depressed individuals experience some impairment in their ability to undertake normal activities and, because of this, depression is a leading cause of disability [1], [3].

Depression among mothers may be of particular concern, both because of the considerable consequences for mothers themselves, but also because of the spillover effects that maternal depression may have on children. Children of depressed mothers have impaired cognitive, behavioral, and health outcomes from infancy through adulthood, and are especially at risk when maternal depression persists [4]–[10]. Depressed mothers often exhibit hostile, negative, or withdrawn behaviors when interacting with their children [6], [11]. Depression may create a stressful living environment, disrupt family routines, or limit mothers’ abilities to parent effectively [11]. It is especially important to understand the prevalence and correlates of depression among mothers of young children, as young children are dependent on their mothers [12]. Understanding depression among unmarried mothers is also important, as this is an increasing demographic group, these mothers may be especially vulnerable, and we know little about the prevalence or correlates of depression for this group [7], [13], [14].

But our descriptive knowledge about maternal depression, especially maternal depression among unmarried mothers, has several limitations. First, much research uses small, nonrepresentative, and homogeneous samples of mothers, resulting in prevalence estimates ranging from 14% to 49% [15]–[21]. Most studies of representative samples of mothers use data that are more than 20 years old [22], [23]. Additionally, studies often focus on the postpartum period and ignore depression when children are in early and middle childhood [22], [24]. Finally, little is known about stability and change in maternal depression, despite research suggesting chronic maternal depression is most detrimental to children [10], [15], [22], [23]. The results of this manuscript, then, make several contributions to our understanding of stability and change in maternal depression, and the findings help inform the development of programs and interventions to diagnose and treat maternal depression.

## Materials and Methods

### Sample

Analyses use data from the Fragile Families and Child Wellbeing Study, a longitudinal survey of nearly 5,000 new and mostly unmarried parents in 20 U.S. cities [25]. Mothers completed a 30- to 40-minute in-person interview at the hospital after the birth of the focal child, between February 1998 and September 2000, and fathers were interviewed as soon as possible after the child’s birth. Mothers and fathers were then interviewed by telephone when their children were approximately 1, 3, 5, and 9 years old. Baseline response rates were 82% for married mothers and 87% for unmarried mothers. Of the 4,898 mothers who participated in the baseline survey, 89%, 86%, 85%, and 74% participated in the 1-, 3-, 5-, and 9-year surveys, respectively [26]. Because unmarried mothers were oversampled, the sample over-represents minority and economically disadvantaged families.

The analytic sample included observations in which the mother completed at least two consecutive surveys (not including the baseline survey, since depression was not measured at baseline) (N = 4,366). Having participated in two consecutive surveys is especially important for the random- and fixed-effects models (described below). Given the majority of mothers participated in two consecutive surveys, there were few differences between the full and analytic samples. Compared to the full sample, mothers in the analytic sample were less likely to be foreign-born. About 15% of mothers in the analytic sample were born outside the United States, compared to 17% of mothers in the full sample (*p*<0.05). Importantly, depression was not associated with attrition, as mothers depressed at the 1-year survey were equally likely as their non-depressed counterparts to participate in each of the following surveys.

### Measures

Maternal depression was assessed when children were 1, 3, 5, and 9 years old (but not at baseline, as mothers had just given birth). The measure of major depressive episode (MDE) came from mothers’ responses to the Composite International Diagnostic Interview-Short Form (CIDI-SF) Version 1.0 [27]. Mothers were asked if, at some time during the past year, they had feelings of dysphoria or anhedonia. Mothers who experienced dysphoria and/or anhedonia for a two-week period most of the day, every day were asked additional questions (about losing interest in things, feeling tired, experiencing a change in weight of at least 10 pounds, having trouble sleeping, having trouble concentrating, feeling worthless, or thinking about death). Those who answered affirmatively to at least one stem question and at least three additional questions were considered depressed. Although limitations to the CIDI-SF exist, it is commonly used in large-scale community surveys to measure depression [28]. Analyses consider the prevalence of depression when children were 1, 3, 5, and 9 years old. Analyses also consider a categorical measure of depression chronicity: no depression (mother reported depression at zero survey waves), intermittent depression (mother reported depression at one or two survey waves), and persistent depression (mother reported depression at three or four survey waves).

The multivariate analyses included a host of control variables. Mother’s race was represented by a series of dummy variables: non-Hispanic white, non-Hispanic black, Hispanic, and non-Hispanic other race. A dummy variable indicated the mother was foreign-born. Additional controls include mother’s age (younger than 25 years, 25 to 34 years, 35 years and older), education (less than high school, high school diploma or GED, post-secondary education), employment in the past week, household income (log), relationship status (married, cohabiting, non-residential romantic relationship, no relationship), and number of children in the household. Finally, analyses adjusted for depression in the child’s father (no depression, intermittent depression, persistent depression), based on fathers’ self-reported responses to the CIDI-SF administered at the 1-, 3-, 5-, and 9-year surveys. In the multinomial logistic regression models, all sociodemographic correlates were measured at the baseline survey (except for employment, which was measured at the 1-year survey). In the random- and fixed-effects logistic regression models, all variables except for race and immigrant status were included as time-varying characteristics (measured at the 1-, 3-, 5-, and 9-year surveys).

### Analysis

The analysis proceeded in three stages. The first stage calculated the prevalence of maternal depression at each of the four survey waves and the prevalence of maternal depression chronicity. Descriptive statistics show sociodemographic correlates, by the prevalence of depression chronicity (no depression, intermittent depression, persistent depression). Mean differences across groups were tested with chi-square tests (for categorical variables) and ANOVA tests (for continuous variables).

The second stage used multinomial logistic regression models to estimate intermittent and persistent depression, both compared to no depression. Model 1 adjusted for the sociodemographic controls and Model 2 included a series of interaction terms between fathers’ depression and mothers’ relationship status with the father (intermittent depression × cohabiting, intermittent depression × non-residential romantic relationship, intermittent depression × no relationship, persistent depression × cohabiting, persistent depression × non-residential romantic relationship, persistent depression × no relationship).

The third and final stage used random- and fixed-effects models to estimate change in depression as a function of change in the covariates. For these models, the data were structured in a parent-wave format where each mother was observed up to four points in time (when children are 1, 3, 5, and 9 years old). The random-effects model captured between- and within-individual variation over time, compared to the more conservative fixed-effects model that captured only within-individual variation. These fixed-effects model allowed for an examination of how *changes* in sociodemographic characteristics between waves were linked to *changes* in depression, net of time-invariant characteristics and time-varying observed characteristics.

Few observations were missing data on covariates, and these observations were preserved using multiple imputation [29]. Both dependent and independent variables were used to impute missing independent variables, but missing dependent variables were not imputed [30]. Findings were robust to listwise deletion. Because observations were drawn from 20 cities, city fixed-effects and clustered standard errors were included in all models. Survey weights were not available for the 9-year survey, but analyses adjusted for all variables used in creating the weights at prior survey waves (race/ethnicity, education, relationship status, and age) [31]. All analyses were performed in Stata/MP 12.1 [32].

## Results

### Prevalence of Depression

Table 1 presents unadjusted descriptive statistics of maternal depression and all demographic characteristics for the analytic sample. Importantly, depression was a common mental health problem among this sample of mostly unmarried mothers. About 16% of mothers were depressed when their children were 1 year old. About 21% were depressed at the 3-year survey, 17% at the 5-year survey, and 17% at the 9-year survey. There was moderate stability in depression at any two consecutive waves, with 52% of mothers depressed at the 1-year survey also depressed at the 3-year survey, 43% of mothers depressed at the 3-year survey also depressed at the 5-year survey, and 44% of mothers depressed at the 5-year survey also depressed at the 9-year survey (descriptives not shown). Importantly, 38% of mothers reported depression at some point during their child’s first nine years. About 31% reported intermittent depression (e.g., depression at one or two survey waves) and 7% reported persistent depression (e.g., depression at three or four survey waves).

In terms of demographic characteristics, the majority of the sample was racial/ethnic minorities. Nearly half of mothers (48%) were non-Hispanic black and more than one-fourth (26%) were Hispanic. About 15% of mothers were born outside the United States, and more than half (54%) were younger than 25 years old when they gave birth to the focal child. Most mothers did not have education beyond high school at baseline, with 34% reporting less than a high school degree and 31% reporting a high school diploma or GED. At baseline, more than three- fifths (61%) of mothers were in a co-residential relationship with the child’s father, and 13% were not in a relationship with the child’s father. More than one-quarter (27%) of children’s fathers reported depression in at least one survey.

**Table 1:** Descriptive Statistics of All Variables Included in Analyses.

	Mean or %	SD	Min	Max
*Maternal depression*				
Depression at 1-year survey	15.5%			
Depression at 3-year survey	20.6%			
Depression at 5-year survey	17.0%			
Depression at 9-year survey	17.4%			
Depression chronicity^a^				
No depression	62.2%			
Intermittent depression	30.7%			
Persistent depression	7.1%			
*Demographic characteristics*				
Race/ethnicity				
Non-Hispanic white	21.6%			
Non-Hispanic black	48.3%			
Hispanic	26.4%			
Non-Hispanic other race	3.7%			
Foreign-born	15.4%			
Age				
Younger than 25 years	53.7%			
25 to 34 years	34.6%			
35 years and older	9.5%			
Education				
Less than high school	33.8%			
High school diploma or GED	30.7%			
Post-secondary education	35.5%			
Employed in past week	53.1%			
Log of household income	9.852	(1.371)	0	11.804
Relationship status with child’s father				
Married	24.4%			
Cohabiting	36.2%			
Non-residential romantic relationship	26.5%			
No relationship	12.9%			
Number of children in household	2.263	(1.305)	1	9
Depression chronicity of child’s father				
No depression	73.0%			
Intermittent depression	24.2%			
Chronic depression	2.9%			
N	4,366			

Note: With two exceptions, all demographic characteristics are measured at baseline. Employed in the past week is measured at the one-year survey, and depression chronicity of the child’s father captures responses from the 1-, 3-, 5-, and 9-year surveys.

a Mothers have intermittent depression if they report depression at one or two of the four survey waves. Mothers have persistent depression if they report depression at three or four of the four survey waves.

### Bivariate Correlates of Maternal Depression Chronicity

Table 2 presents unadjusted sociodemographic characteristics, by maternal depression chronicity (no depression, intermittent depression, persistent depression). To begin with, the descriptive statistics showed statistically significant race/ethnic differences in maternal depression chronicity. Non-Hispanic white and non-Hispanic black mothers were less likely to report no depression or intermittent depression, compared to persistent depression. Hispanic and other race mothers were less likely to report persistent depression, compared to no depression or intermittent depression (*p = *0.002). About 8% of mothers who reported persistent depression were foreign-born, compared to 13% of mothers who reported intermittent depression and 19% of mothers who reported no depression (*p = *0.000). Mothers who reported intermittent or persistent depression, compared to those who reported no depression, had lower educational attainment at baseline (*p = *0.001). They were also less likely to be employed at the 1-year survey (*p* = 0.000) and had lower household incomes (*p = *0.000). Relationship status at baseline was also correlated with depression. For example, 27% of mothers who reported no depression were married at baseline, compared to 19% with intermittent depression and 19% with persistent depression. Similarly, 12% of mothers who reported no depression were no longer in a relationship with the child’s father at baseline, compared to 14% with intermittent depression and 18% with persistent depression (*p = *0.000). Mothers with intermittent and persistent depression had more children, on average, than their counterparts with no depression (*p = *0.045). There was also an association between mothers’ and fathers’ depression. More than three-quarters (77%) of mothers with no depression shared children with fathers who reported no depression, compared to 70% of mothers with intermittent depression and 59% of mothers with persistent depression (*p = *0.000).

**Table 2:** Demographic Characteristics, by Maternal Depression Chronicity.

	Maternal depression chronicity^a^			
	No depression	Intermittent depression	Persistentdepression	*p*
Race/ethnicity				0.002
Non-Hispanic white	20.9%	20.6%	24.9%	
Non-Hispanic black	45.8%	50.4%	53.7%	
Hispanic	29.0%	25.6%	19.1%	
Non-Hispanic other race	4.3%	3.4%	2.3%	
Foreign-born	18.9%	13.2%	8.0%	0.000
Age				0.005
Younger than 25 years	51.4%	56.9%	57.9%	
25 to 34 years	35.9%	33.6%	30.5%	
35 years and older	10.4%	7.5%	10.0%	
Education				0.000
Less than high school	32.2%	39.0%	33.9%	
High school diploma or GED	30.5%	30.2%	30.3%	
Post-secondary education	37.3%	30.8%	35.8%	
Employed in past week	55.3%	48.4%	51.3%	0.000
Log of household income	9.917	9.740	9.629	0.000
Relationship status with child’s father				0.000
Married	27.4%	19.2%	19.0%	
Cohabiting	35.2%	38.0%	40.8%	
Non-residential romantic relationship	25.3%	28.6%	22.5%	
No relationship	12.1%	14.3%	17.7%	
Number of children in household	2.224	2.320	2.325	0.045
Depression chronicity of child’s father				0.000
No depression	76.9%	69.8%	58.9%	
Intermittent depression	20.8%	26.6%	37.9%	
Persistent depression	2.3%	3.6%	3.2%	
N	2,715	1,340	311	

Note: With two exceptions, all demographic characteristics are measured at baseline. Employed in the past week is measured at the 1-year survey, and depression chronicity of the child’s father captures responses from the 1-, 3-, 5-, and 9-year surveys. Chi-square tests or ANOVA tests, depending on the distribution of the independent variable, compare differences across groups.

a Mothers have intermittent depression if they report depression at one or two of the four survey waves. Mothers have persistent depression if they report depression at three or four of the four survey waves.

### Multinomial Logistic Regression Models Estimating Intermittent and Persistent Depression

Table 3 presents results from multinomial logistic regression models estimating depression chronicity. The first two models estimated the probability of mothers reporting intermittent depression. The reference group was mothers reporting no depression. In Model 1, which adjusted for all control variables, there were no race/ethnic differences, suggesting mothers of all race/ethnic groups were equally likely to report intermittent depression compared to no depression. Similarly, both native- and foreign-born mothers were equally likely to report intermittent depression compared to no depression. Socioeconomic characteristics were, however, related to maternal depression. Mothers with a high school diploma or GED, compared to mothers who did not graduate high school, were less likely to report intermittent depression than no depression (OR = 0.85, CI = 0.74, 0.99). Employed mothers were less likely to report intermittent depression than no depression (OR = 0.80, CI = 0.72, 0.90), and household income was inversely associated with intermittent depression (OR = 0.96, CI = 0.92, 1.00). Baseline relationship status was also associated with a greater likelihood of reporting intermittent depression compared to no depression. Compared to mothers married at baseline, mothers cohabiting at baseline had 1.39 times the odds of reporting intermittent depression (CI = 1.11, 1.74), mothers in nonresidential romantic relationships had 1.36 times the odds of reporting intermittent depression (CI = 1.06, 1.76), and mothers not in a relationship had 1.43 times the odds of reporting intermittent depression (CI = 1.09, 1.87). Finally, Model 1 documented an association between fathers’ and mothers’ depression chronicity. Mothers who shared children with men reporting intermittent depression, compared to no depression, were more likely to report intermittent depression themselves (OR = 1.25, CI = 1.06, 2.12). Persistent depression among fathers was also positively associated with intermittent depression among mothers, though this coefficient did not reach statistical significance, likely because of the few number of fathers who fell into this category.

**Table 3:** Multinomial Logistic Regression Models Estimating Maternal Depression Chronicity.

	Intermittent depression vs. no depression						Persistent depression vs. no depression					
	Model 1			Model 2			Model 1			Model 2		
	OR	95% CI		OR	95% CI		OR	95% CI		OR	95% CI	
Race/ethnicity												
Non-Hispanic White (reference)	–	–		–	–		–	–		–	–	
Non-Hispanic Black	0.96	(0.79, 1.17)		0.96	(0.78, 1.17)		0.89	(0.65, 1.23)		0.89	(0.64, 1.22)	
Hispanic	0.87	(0.65, 1.16)		0.86	(0.64, 1.16)		0.61	(0.39, 0.98)	*	0.61	(0.38, 0.97)	*
Non-Hispanic other race	1.01	(0.67, 1.52)		1.02	(0.68, 1.53)		0.68	(0.29, 1.58)		0.69	(0.29, 1.62)	
Foreign-born	0.77	(0.54, 1.09)		0.77	(0.54, 1.09)		0.57	(0.31, 1.04)	∧	0.57	(0.31, 1.04)	∧
Age												
Younger than 25 years (reference)	–	–		–	–		–	–		–	–	
25 to 34 years	1.06	(0.90, 1.24)		1.06	(0.91, 1.24)		0.90	(0.71, 1.15)		0.91	(0.71, 1.15)	
35 years and older	0.85	(0.65, 1.11)		0.85	(0.65, 1.10)		1.06	(0.63, 1.79)		1.05	(0.62, 1.78)	
Education												
Less than high school (reference)	–	–		–	–		–	–		–	–	
High school diploma or GED	0.85	(0.74, 0.99)	*	0.85	(0.74, 0.98)	*	0.93	(0.61, 1.43)		0.93	(0.61, 1.43)	
Post-secondary education	0.83	(0.67, 1.04)		0.83	(0.67, 1.04)		1.14	(0.81, 1.60)		1.14	(0.82, 1.59)	
Employed in past week	0.80	(0.72, 0.90)	***	0.80	(0.72, 0.90)	***	0.86	(0.65, 1.14)		0.86	(0.65, 1.14)	
Log of household income	0.96	(0.92, 1.00)	∧	0.96	(0.93, 1.00)	∧	0.88	(0.82, 0.95)	**	0.88	(0.81, 0.95)	**
Relationship status with child’s father												
Married (reference)	–	–		–	–		–	–		–	–	
Cohabiting	1.39	(1.11, 1.74)	**	1.47	(1.11, 1.96)	**	1.53	(0.88, 2.68)		1.96	(1.10, 3.49)	*
Non-residential romantic relationship	1.36	(1.06, 1.76)	*	1.58	(1.14, 2.18)	**	0.98	(0.49, 1.97)		1.35	(0.61, 2.96)	
No relationship	1.43	(1.09, 1.87)	**	1.67	(1.19, 2.33)	**	1.64	(0.92, 2.91)	∧	2.38	(1.27, 4.46)	**
Number of children in household	1.03	(0.98, 1.47)		1.02	(0.98, 1.07)		1.03	(0.93, 1.14)		1.03	(0.94, 1.14)	
Depression chronicity of child’s father												
No depression (reference)	–	–		–	–		–	–		–	–	
Intermittent depression	1.25	(1.06, 2.12)	**	1.70	(1.14, 2.52)	**	1.92	(1.55, 2.36)	***	3.60	(1.93, 6.73)	***
Persistent depression	1.37	(0.89, 1.09)		2.12	(0.80, 5.62)		1.33	(0.64, 2.76)		3.32	(0.78, 14.21)	
Depression chronicity of child’s father * relationship status												
Intermittent depression * cohabiting				0.78	(0.50, 1.20)					0.51	(0.22, 1.19)	
Intermittent depression * non-residential romantic relationship				0.60	(0.33, 1.08)	∧				0.41	(0.19, 0.90)	*
Intermittent depression * no relationship				0.62	(0.38, 1.02)	∧				0.41	(0.17, 0.99)	*
Persistent depression * cohabiting				0.73	(0.25, 2.09)					0.40	(0.11, 1.48)	
Persistent depression * non-residential romantic relationship				0.58	(0.22, 1.50)					0.37	(0.05, 2.61)	
Persistent depression * no relationship				0.45	(0.13, 1.53)					0.11	(0.80, 117.57)	
Intercept	−0.40			−0.48			−1.09			−1.320		
Log likelihood	−3615			−3605			−3615			−3605		
N	4,366			4,366			4,366			4,366		

Note: With two exceptions, all demographic characteristics are measured at baseline. Employed in the past week is measured at the 1-year survey, and depression chronicity of the child’s father captures responses from the 1-, 3-, 5-, and 9-year surveys. OR = odds ratio; CI = confidence interval.

∧ p<0.10,

* p<0.05,

** p<0.01,

*** p<0.001.

a Mothers have intermittent depression if they report depression at one or two of the four survey waves. Mothers have persistent depression if they report depression at three or four of the four survey waves.

Interaction terms between fathers’ depression and mothers’ relationship status with the father were added to Model 2. These interaction terms suggest that fathers’ intermittent depression was less strongly associated with mothers’ intermittent depression, compared to no depression, when mothers and fathers were in a nonresidential romantic relationship (OR = 0.60, CI = 0.33, 1.08) or no relationship (OR = 0.62, CI = 0.38, 1.02) compared to being married. The pattern of results is perhaps more striking – and certainly easier to interpret – by considering coefficients (not presented) instead of odds ratios. For example, the coefficient for fathers’ intermittent depression (0.53) was nearly wiped out by the coefficients for the intermittent depression × non-residential romantic relationship interaction (-0.52) and for the intermittent depression × no relationship interaction (−0.45). Interactions between fathers’ persistent depression and relationship status did not reach statistical significance, likely due to small sample sizes, but the point estimates went in the expected direction.

The final two models in Table 3 estimate the probability of mothers reporting persistent depression. Again, the reference group was mothers reporting no depression and Model 1 included all control variables. Importantly, the factors associated with intermittent depression were not necessarily the same factors associated with persistent depression. For one, though not associated with intermittent depression, race/ethnicity and immigrant status were associated with persistent depression. Hispanic mothers, compared to their non-Hispanic White counterparts, were less likely to report persistent depression than no depression (OR = 0.61, CI = 0.39, 0.98). Foreign-born mothers were also less likely to report persistent depression, compared to no depression, than their native-born counterparts, though this coefficient was only marginally significant (OR = 0.57, CI = 0.31, 1.04). Household income was associated with persistent depression, as mothers with higher household incomes were less likely to report persistent depression than no depression (OR = 0.88, CI = 0.82, 0.95). Additionally, mothers not in a relationship at baseline, compared to those married at baseline, had a greater likelihood of reporting persistent depression than no depression (OR = 1.64, CI = 0.92, 2.91). Similar to estimates predicting intermittent depression, mothers who shared children with depressed men – especially intermittently depressed men – were more likely to report persistent depression than no depression (OR = 1.92, 1.55, 2.36).

Model 2 includes interaction terms between fathers’ depression and mothers’ relationship status with the father. Interaction terms between relationship status and fathers’ depression, showed that the relationship between fathers’ intermittent depression and mothers’ persistent depression was weaker when mothers and fathers were in a nonresidential romantic relationship (OR = 0.41, CI = 0.19, 0.90) or not in a relationship at baseline (OR = 0.41, CI = 0.17, 0.99) compared to being married. Again, the pattern of results is easier to interpret when considering coefficients (not presented). In estimating persistent maternal depression, the coefficient for fathers’ intermittent depression (1.28) was nearly wiped out by the coefficients for the intermittent depression × non-residential romantic relationship interaction (−0.89) and for the intermittent depression × no relationship interaction (−0.88). Interactions between fathers’ persistent depression and relationship status did not reach statistical significance, likely due to the small number of fathers who fell into this category and the large confidence intervals, but the point estimates were in the expected direction.

Importantly, there was only one statistically significant difference in the correlates of intermittent and persistent depression (coefficients not shown). Fathers’ intermittent depression, compared to no depression, was associated less with mothers’ intermittent depression than persistent depression (OR = 0.65, CI = 0.51, 0.83).

### Random- and Fixed-Effects Logistic Regression Models Estimating Change in Depression

In Table 4, the random- and fixed-effects logistic regression models took into account all sociodemographic characteristics included in the previous multivariate models, but included time-varying characteristics whenever possible (e.g., age, education, employment, household income, relationship status, number of children in household, fathers’ depression). Turning to the first column, which presents the random-effects model, the correlates of change in maternal depression were similar to the correlates of maternal depression chronicity. Hispanic mothers, compared to white mothers, were less likely to report depression (OR = 0.71, CI = 0.53, 0.91), and foreign-born mothers were less likely than native-born mothers to report depression (OR = 0.68, CI = 0.53, 0.89). Additionally, employed mothers reported less depression than mothers not working (OR = 0.67, CI = 0.59, 0.75), and household income was negatively associated with depression (OR = 0.93, CI = 0.89, 0.97). Mothers in a nonresidential romantic relationship or no relationship with their child’s father, compared to married mothers, had a greater odds of depression (OR = 1.49, CI = 1.23, 1.81; OR = 1.84, CI = 1.55, 2.18). Fathers’ depression was associated with a greater odds of depression among mothers (OR = 1.37, CI = 1.14, 1.64). The association between changes in relationship status and changes in maternal depression did not vary by changes in fathers’ depression (not presented).

**Table 4:** Random- and Fixed-Models Estimating Change in Maternal Depression.

	Random-effects model			Fixed-effects model		
	OR	95% CI		OR	95% CI	
Race/ethnicity						
Non-Hispanic white (reference)	–	–		–	–	
Non-Hispanic black	0.85	(0.69, 1.04)		–	–	
Hispanic	0.71	(0.56, 0.91)	**	–	–	
Non-Hispanic other race	0.82	(0.51, 1.32)		–	–	
Foreign-born	0.68	(0.53, 0.89)	**	–	–	
Age						
Younger than 25 years (reference)	–	–		–	–	
25 to 34 years	1.03	(0.91, 1.18)		1.01	(0.87, 1.17)	
35 years and older	0.96	(0.80, 1.16)		1.01	(0.78, 1.30)	
Education						
Less than high school (reference)	–	–		–	–	
High school diploma or GED	0.90	(0.73, 1.11)		1.14	(0.74, 1.78)	
Post-secondary education	0.90	(0.75, 1.08)		0.92	(0.65, 1.32)	
Employed in past week	0.67	(0.59, 0.75)	***	0.81	(0.71, 0.94)	**
Log of household income	0.93	(0.89, 0.97)	***	0.97	(0.92, 1.02)	
Relationship status with child’s father						
Married (reference)	–	–		–	–	
Cohabiting	1.10	(0.94, 1.30)		1.03	(0.84, 1.26)	
Non-residential romantic relationship	1.49	(1.23, 1.81)	***	1.21	(0.97, 1.52)	∧
No relationship	1.84	(1.55, 2.18)	***	1.58	(1.28, 1.96)	***
Number of children in household	1.02	(0.97, 1.07)		1.01	(0.94, 1.07)	
Depression in child’s father	1.37	(1.14, 1.64)	**	1.22	(0.99, 1.49)	∧
Log likelihood	−6,609			−2,041		
Person-year observations	15,758			5,555		
N	4,366			1,496		

Note: OR = odds ratio; CI = confidence interval.

∧ p<0.10,

* p<0.05,

** p<0.01,

*** p<0.001.

The fixed-effect model, presented in the second column, shows only employment, relationship status, and depression in the child’s father were statistically significant independent predictors of change in maternal depression. Becoming employed between survey waves was associated with a lower likelihood of becoming depressed between survey waves (OR = 0.81, CI = 0.71, 0.94). Additionally, mothers who moved into no relationship between survey waves had 1.58 times the odds of becoming depressed between survey waves (CI = 1.28, 1.96). Further, changes in fathers’ depression was positively associated with changes in depression (OR = 1.22, CI = 0.99, 1.49). Changes in other time-varying characteristics were not associated with changes in depression, suggesting that pre-existing, stable characteristics may be partly responsible for the statistically significant associations shown in the random-effects models.

## Discussion

This manuscript uses recent, longitudinal data from the Fragile Families and Child Wellbeing Study, a sample of mostly unmarried parents, to estimate the prevalence and correlates of maternal depression. Results show that, at any given survey wave, between 16% and 21% of mothers reported depression. Nearly two-fifths (38%) reported depression at least once during the eight-year period. About 31% reported intermittent depression (e.g., depression at one or two survey waves) and 7% reported persistent depression (e.g., depression at three or four survey waves). These prevalence estimates are higher than estimates of the general population and lower than estimates found in samples of disadvantaged mothers [1], [16]–[18]. They are consistent with other research on representative samples of mothers, though it is difficult to make comparisons because most representative studies use the Center for Epidemiologic Studies Depression Scale (CES-D) to measure depression (instead of the CIDI-SF) [22]–[23], [33].

A second goal of this paper was to estimate the correlates of maternal depression. Consistent with previous research, employment and relationship status are among the sociodemographic characteristics most robustly associated with both stability and change maternal depression [1], [24], [33]–[37]. The association between relationship status and maternal depression is complicated, though, as depression in the child’s father was also robustly associated with both stability and change in maternal depression if the parents were living together. This is consistent with other research on assortative mating by mental health [38] and suggests that simply being in a marital relationship may not be enough to ameliorate depression among mothers (and, instead, that partner characteristics are important). Other demographic characteristics – including education and employment – were associated with intermittent depression but not persistent depression, which is consistent with research suggesting differential causes of depression onset and depression persistence [23], [37].

Taken together, these findings make several contributions to the existing literature on maternal depression. First, the estimates presented use data from a recent, large, longitudinal and heterogeneous sample of a group most at risk of maternal depression. This is in contrast to much existing research that relies on outdated or non-representative samples. Second, the estimates consider depression among mothers over an eight-year time period that spans both early and middle childhood. Third, by examining both stability and change in maternal depression over this eight-year period, as well as using rigorous fixed-effects models that examine within-person changes in maternal depression, these findings add to a literature on the correlates of maternal depression that is often limited to considering depression at one point in time.

Though these analyses provide a comprehensive portrait of the prevalence and correlates of maternal depression, several limitations exist. First, because unmarried mothers were oversampled, the sample is over-representative of minority and economically disadvantaged mothers. Though these results cannot be generalized to all mothers, they provide a comprehensive understanding of an increasing, vulnerable demographic group [7], [13]. Unmarried mothers and their children have been the focus of increasing research and policy initiatives and, indeed, some suggest that such family arrangements lead to increasing inequality and the reproduction of poverty [39], [40].

Additional limitations exist. The dichotomous measure of depression makes it impossible to consider mothers who exhibited some symptoms of depression but did not fit the diagnostic criterion for depression [41]. Similarly, for mothers classified as depressed, the dichotomous measure did not allow for an examination of heterogeneity in depression severity, despite the fact that children may suffer most when mothers are severely depressed [42]. Additionally, despite the fact the data span an eight-year time period, yearly measures of depression were only assessed four times. For example, a mother who experienced a short-lived bout of depression between the 1- and 3-year surveys would only fit the diagnostic criterion for yearly depression if that depression occurred in the year prior to the 3-year survey. Given this, it is likely the true prevalence of maternal depression is underreported. Finally, the analyses cannot demonstrate a causal relationship between the sociodemographic characteristics and maternal depression. Certainly, socioeconomic status, relationship status, and fathers’ depression are likely both causes and consequences of maternal depression, and these analyses simply lay the groundwork for future research to understand these complicated, likely bi-directional relationships.

Despite these limitations, the findings have important implications for public health professionals. To begin with, they suggest a large number of mothers suffer from depression when their children are in early and middle childhood. But depressed mothers – at least the mostly unmarried mothers in this sample – are not necessarily receiving treatment for their condition. In fact, supplemental analyses (not presented) show that 80% of mothers depressed at the 3-year survey do not receive counseling or therapy for depression and 86% are not taking anti-depressants. Screening procedures by primary care physicians and pediatricians may help identify and treat depressed mothers. Similarly, this group of mostly unmarried mothers may face particular barriers that prevent them from seeking and receiving treatment (e.g., impediments to finding child care, difficulty affording co-payments or prescriptions) [43]. Researchers and practitioners should work to identify and eliminate these barriers.

Additionally, these findings attest to the salience of socioeconomic status as a correlate of maternal depression. Understanding this and other risk factors associated with depression may help physicians identify mothers who may especially benefit from treatment. Given that socioeconomic status is amenable to policy interventions such as anti-poverty programs that promote economic self-sufficiency, effective prevention and treatment strategies may include targeting such correlates of depression [44]. Improving economic self-sufficiency may also increase the probability of marriage [45], an additional correlate of maternal depression. However, given that fathers’ depression plays an important role in maternal depression, these results suggest that policymakers concerned with marriage promotion among economically disadvantaged couples must consider parents’ mental health [24]. Given the large social consequences of maternal depression, not least of which is impaired wellbeing among children of depressed mothers, prevention and treatment of maternal depression should be an imperative for public health professionals [10].
